# Chicago Beef

**Published:** 1884-08

**Authors:** 


### CHICAGO BEEF.

NORTHERN people who visited Charleston or Savannah used to read with some surprise at hotel dinner-tables, upon their bills of fare : "Roast sirloin of beef, from Fulton market."

We have had the pleasure of eating a portion of such a sirloin from Fulton market, which had made the four days' voyage packed in ice. The pleasure was not great, since it did not improve Fulton market beef to freeze it solid in an ice-chest in New York, and thaw it out in a Charleston kitchen. That was long ago, and people had not learned how to get or how to treat good beef.

Fulton market still stands, and fine beef is still sold therein; but a new system prevails by which beef is procured and distributed all over this broad land. Let us suppose that the reader is seated at breakfast at a sea-side hotel on the coast of Maine, and that he has ordered beefsteak as his principal viand.

That steak, tender as it is, has a long, eventful history. Four years ago, the creature of which it recently formed a part, was a calf frisking on the prairies of western Texas, where it passed the first two years of its life wandering with a herd on a ranch possibly fifty miles or more in circuit.

Then it was slowly driven northward a thousand miles to a greater ranch in Wyoming, Montana, or Colorado, where the climate is more favorable to the production of good beefsteak.

*Driven* is not quite the word to describe the slow drifting of a great drove of Texas cattle, feeding as it goes, and making twelve miles a day without knowing it. These cattle are nearly three months on the way, and if they are "handled" skilfully, they arrive at their cooler range in as good condition as when they left hot Texas. Perhaps, in better.

Invigorated by the bracing air and nutritious grass of Wyoming or Montana, they pass two years in peaceful self-improvement, developing into sleek, fat steers, worth thirty-five dollars each as they run.

So far, all has gone well with them; but now comes a sharp, short period of trial. The owner and his assistants drive a thousand of the four-year-olds to the nearest railroad station, probably many miles distant, where they are quickly loaded into a train of fifty cattle-cars, and conveyed, in about five days, to Chicago. They *ought* to be fed and watered every eight hours. Some are; but not all.

And here we will pause to make an observation. If your steak is good and tender, and altogether as it should be, take comfort in the thought that the animal from which it came enjoyed a happy existence. He was fairly and gently dealt with from his frisky calfhood to his last hours. If he has been over-driven, under-fed or harshly treated, he has his revenge after his death in the disgust and indigestion his toughness causes.

Arrived at Chicago, he is fed, watered, rested, sold, shot, dressed, chilled, and placed in refrigerating cars, and sent to an agency in an Eastern city, whence he is taken to your summer resort in Maine and a small part of him cooked for the reader's breakfast. It is thus that Chicago supersedes Fulton Market.—[*Youth's Companion.*

Doctor (engaged six months after the death of his first wife, soliloquizing over a letter)—"This is better. She addresses me as 'darling duck.' My first wife used to speak of me as 'old quack'."

